# Therapeutic Role of Ursolic Acid on Ameliorating Hepatic Steatosis and Improving Metabolic Disorders in High-Fat Diet-Induced Non-Alcoholic Fatty Liver Disease Rats

**DOI:** 10.1371/journal.pone.0086724

**Published:** 2014-01-29

**Authors:** Songtao Li, Fanyu Meng, Xilu Liao, Yemei Wang, Zongxiang Sun, Fuchuan Guo, Xiaoxia Li, Man Meng, Ying Li, Changhao Sun

**Affiliations:** Department of Nutrition and Food Hygiene, Harbin Medical University, Harbin, Heilongjiang province, P. R. China; CIMA. University of Navarra, Spain

## Abstract

**Background:**

Non-alcoholic fatty liver disease (NAFLD) is one of the most prevalent liver diseases around the world, and is closely associated with obesity, diabetes, and insulin resistance. Ursolic acid (UA), an ubiquitous triterpenoid with multifold biological roles, is distributed in various plants. This study was conducted to investigate the therapeutic effect and potential mechanisms of UA against hepatic steatosis in a high-fat diet (HFD)-induced obese non-alcoholic fatty liver disease (NAFLD) rat model.

**Methodology/Principal Findings:**

Obese NAFLD model was established in Sprague-Dawley rats by 8-week HFD feeding. Therapeutic role of UA was evaluated using 0.125%, 0.25%, 0.5% UA-supplemented diet for another 6 weeks. The results from both morphologic and histological detections indicated that UA significantly reversed HFD-induced hepatic steatosis and liver injury. Besides, hepatic peroxisome proliferator-activated receptor (PPAR)-α was markedly up-regulated at both mRNA and protein levels by UA. Knocking down PPAR-α significantly inhibited the anti-steatosis role of UA *in vitro*. HFD-induced adverse changes in the key genes, which participated in hepatic lipid metabolism, were also alleviated by UA treatment. Furthermore, UA significantly ameliorated HFD-induced metabolic disorders, including insulin resistance, inflammation and oxidative stress.

**Conclusions/Significance:**

These results demonstrated that UA effectively ameliorated HFD-induced hepatic steatosis through a PPAR-α involved pathway, via improving key enzymes in the controlling of lipids metabolism. The metabolic disorders were accordingly improved with the decrease of hepatic steatosis. Thereby, UA could be a promising candidate for the treatment of NAFLD.

## Introduction

Non-alcoholic fatty liver disease (NAFLD) encompasses a cluster of liver disorders, ranging from accumulation of fat in liver (steatosis) to nonalcoholic steatohepatitis (necrosis and inflammation), with some people ultimately progressing to fibrosis, cirrhosis and liver failure. Because of its high prevalence in conjunction with obesity, diabetes, and insulin resistance, NAFLD is being increasingly appreciated as a hepatic manifestation of the metabolic syndrome and represents a major cause of liver-related morbidity and mortality [1]–[3].

The pathogenesis of NAFLD is still unclear, although several hypotheses exist. One generally accepted theory is the “two-hit” hypothesis [4], wherein the first hit involves the progression of hepatic steatosis and the impairment in the key enzymes in regulating triglyceride (TG) and free fatty acids (FFA) metabolism [5]–[7], rendering the liver more susceptible to a second undefined hit, resulting in more severe liver damage. Although simple steatosis was originally thought to be a pathologically inert histological change, there is now convincing evidence that fat accumulation in the liver plays a critical role not only in the initiation, but also in the progression of NAFLD. For instance, steatotic livers are more sensitive to hepatotoxicity caused by agents such as endotoxin [8]. Furthermore, the degree of TG deposition is predictive of the severity of later stages of NAFLD (i.e., fibrosis and cirrhosis) [9]. Therefore, improvement of hepatic lipid metabolism and attenuation of fat accumulation have been shown to have therapeutic potential in preventing the progression of NAFLD and alleviating insulin resistance, inflammation and oxidative stress.

However, there is currently no Food and Drug Administration (FDA) approved treatment for NAFLD or nonalcoholic steatohepatitis. NAFLD is considered reversible through modifying lifestyle [10], including weight loss by a combination of decreased caloric intake and increased physical activity. But in practice, it is difficult to achieve and even more difficult to maintain for most patients. Therefore, searching for the safe compound with high preventive and/or therapeutic efficacies is urgently needed.

Ursolic acid (UA), a natural pentacyclic triterpenoid carboxylic acid, is widely distributed in various plants, and has become an integral part of human diet [11]. Increasing evidence, both *in vitro* and *in vivo*, suggests that UA has a variety of biological roles, including anti-inflammatory, anti-oxidative, anti-mutagenic, anti-carcinogenic, anti-microbial, anti-atherosclerotic, and anti-hyperlipidemic effects [12], [13]. Our previous study has shown that UA stimulated TG hydrolysis via activating adipose triglyceride lipase (ATGL) and hormone-sensitive lipase (HSL), the rate-limiting enzymes for lipolysis in primary-cultured adipocytes [14]. In C57BL/6 mice, administrating UA into the high-fat diet (HFD) significantly prevented HFD-induced increase of body weight, visceral fat accumulation, and ameliorated glucose intolerance [15]–[18]. Moreover, it is well accepted that NAFLD is particularly associated with the presence and severity of obesity [19], [20]. The imbalance in energy intake and its combustion is commonly regarded as the link between NAFLD and obesity via aggravating lipid storage. We, therefore, hypothesized that UA could improve NAFLD with obesity through regulating lipid metabolism. Although the effect of UA on NAFLD has been studied by few scientific groups [15]–[18], only preventive role was investigated in these studies, because UA was administrated together with the HFD, and no published report has comprehensively clarified the therapeutic role in anti-NAFLD with rat. Therefore, the current study was aimed at demonstrating the therapeutic effect of UA against hepatic steatosis, insulin resistance, inflammation and oxidative stress in a HFD-induced obese NAFLD rat model, and further addressing its potential mechanisms.

## Materials and Methods

### Ethics Statement

All protocols in this study were approved by the Medical Ethics Committee of Harbin Medical University (Habrin, China) and were performed in accordance with the National Institutes of Health regulations for the care and use of animals in research.

### Animal Care and Experimental Protocol

Male Sprague-Dawley rats (150–160 g, 5 weeks, Beijing Vital River Laboratory Animal Technology Co., Ltd.) were maintained at a 12∶12 h light: dark cycle, and given water ad libitum. Animals were housed in cages individually in an environmentally controlled room at 21±2.0°C, and 50±5% humidity.

### Establishment of Rat Obese NAFLD Model

After one week adaptation, rats (6 weeks of age) were randomly divided into normal-fat diet (NFD) group (n = 13) and HFD group (n = 70) by weight, and fed for 8 weeks, respectively (Diet formula was showed in Table 1). Forty four rats in the HFD group, whose body weight was larger than the mean body weight plus one fold of the standard deviation of the NFD group, were designated as obese NAFLD rats; the other rats in the HFD group were designated as obese resistance (n = 26) and excluded from this study. Hepatic TG content and weight in rats from obese NAFLD (n = 4) and NFD group (n = 3) were detected to confirm the establishment of this model.

**Table 1:** Diet formula.

Diet	NFD (kcal%)	HFD (kcal%)
Protein	20	20
Carbohydrates	70	35
Fat	10	45
Ingredients, g		
Casein (80 mesh)	200	200
L-Cystine	3	3
Corn Starch	315	72.8
Maltodextrin 10	35	100
Sucrose	350	172.8
Cellulose, BW200	50	50
Soybean Oil	25	25
Lard	20	177.5
Mineral mix S10026	10	10
DiCalcium Phosphate	13	13
Calcium Carbonate	5.5	5.5
Potassium Citrate, 1 H2O	16.5	16.5
Vitamin Mix V10001	10	10
Choline Bitartrate	2	2

### UA Treatment and Sample Collection

Obese NAFLD rats (n = 40) were randomly sorted into 4 groups in equation by weight, and fed HFD, 0.125% UA (L-UA), 0.25% UA (M-UA), and 0.5% UA (H-UA) supplemented HFD for 6 weeks, respectively. Rats in control group (n = 10) were maintain with NFD. UA (98% purity) was bought from Zelang Medical Technology Co., LTD (Nanjing, China).

Food intake and body weight were measured daily and weekly, respectively. At week 14, rats were anaesthetised with sodium pentobarbital anaesthesia (30mg/kg body weight, intraperitoneal injection) and blood was collected from the abdominal aorta. Serum was obtained and stored at −80°C until analysis. Tissues, including liver, skeletal muscle, perirenal and epididymal adipose tissue were removed, weighted and snap-frozen in liquid nitrogen, and stored at −80°C immediately.

### Cell Culture

A human normally hepatic immortal cell line HL-7702 obtained from Shanghai Cell Biology Institute, Chinese Academy of Sciences [21], [22], was used in the experiments. The cells were cultured in RPMI 1640 medium supplemented with 10% fetal bovine serum, antibiotics (100 U/ml penicillin, 100 mg/ml streptomycin) at 37°C in a humidified atmosphere of 5% CO_2_ and 95% air. Oleic acid (OA) and fenofibrate were purchased from Sigma.

### Measurement of Serum Parameters

Serum glucose (GLU), TG, total cholesterol (TC), high-density lipoprotein cholesterol (HDL-C), low-density lipoprotein cholesterol (LDL-C), alanine aminotransferase (ALT), aspartate aminotransferase (AST), and FFA levels were determined with an automatic analyzer (HITACHI-7100,Japan; all kits were supplied by Roche). Serum inflammatory factors and hormones were measured by using various commercially available ELISA kits for rat. Specifically, for tumor necrosis factor (TNF-α), monocyte chemotactic protein-1 (CCL2/MCP-1), leptin, and adiponectin levels measurements, the Enzo Life Sciences ELISA kit was used (Enzo Life Sciences Inc., NY); for serum interleukin (IL)-6 levels, the R&D Systems ELISA kit (R&D Systems Europe, Abingdon, UK) and for insulin levels the BioVendor ELISA kit (Shibayagi Co., Ltd., Gunma, Japan). Serum malondialdehyde (MDA), superoxide dismutase (SOD), catalase (CAT) and glutathione peroxidase (GPx) were measured with commercial kits using enzymatic methods (Jiancheng Technology, Nanjing, China).

### Histological Examination

At the time of killing, small pieces of liver were fixed immediately in 10% buffered formalin. After paraffin embedding, 5 µm sections were deparaffinized in xylene and were rehydrated through a series of decreasing concentrations of ethanol. Sections were stained with hematoxylin and eosin (HE). Alternatively, portions of fresh liver were flash-frozen and cryostat sections were cut and prepared for staining with Oil Red O. Photomicrographs were taken on a Nikon Eclipse E600 microscope (Fryer, Cincinnati, OH).

### RNA Extraction and Quantitative RT-PCR

Total RNA from rat liver tissue or HL-7702 cells were extracted using TRIzol reagent (Invitrogen, CA). Real-time PCR was performed with the SYBR Green PCR Master Mix and a 7500 FAST Real-time PCR System (Applied Biosystems), using the primers shown in table 2. All reactions were performed at least in triplicate.

**Table 2:** Primer sequence for quantitative real-time PCR.

Gene	Sequence
*β-actin*	F: 5′-AGGGAAATCGTGCGTGAC-3′
	R: 5′-CGCTCATTGCCGATAGTG-3′
*PPAR-α*	F: 5′-TCATACTCGCAGGAAAGACT-3′
	R: 5′-ACCTCTGCCTCCTTGTTTTC-3
*FAT/CD36*	F: 5′-CGGCGATGAGAAAGCAGAA-3′
	R: 5′-CCAGGCCCAGGAGCT TTATT-3′
*DGAT1*	F: 5′-TTTCTGCTACGGCGGGTTCTTGAG-3′
	R: 5′-ACCGGTTGCCCAATGATGAGTGTC-3′
*DGAT2*	F: 5′-GGAGGCCACCGAAGTTAGCAAGAA-3'
	R: 5′-AGCCCCCAGGTGTCAGAGGAGAAG-3′
*ATGL*	F: 5′-CACTTTAGCTCCAAGGATGA-3′
	R: 5′-TGGTTCAGTAGGCCATTCCT-3′
*HSL*	F: 5′-ACATGGCCTTCTTCTCAAGC-3′
	R: 5′-TCATGGGATTTGGAGGTCTG-3′
*CPT-1*	F: 5′-GCCCATGTTGTACAGCTTCCA-3′
	R: 5′-AGTCTTCTTCCTTCATCAGTGGC-3′
*SREBP-1c*	F: 5′-GCCATCGACTACATCCGCTT-3′
	R: 5′-CAGGTCTTTCAGTGATTTGCTTTT-3′
*FAS*	F: 5′-TCTCTGGTGGTGTCTACATTTCG-3′
	R: 5′-GCAGGATAGCACTCTCAGACAG-3′
*ACC*	F: 5′-TTTTCGATGTCCTCCCAAACTTTT-3′
	R: 5′-GCTCATAGGCGATATAAGCTCTTC-3′
*TNF-α*	F: 5′-TGTCTCAGCCTCTTCTCATT-3′
	R: 5′-AGATGATCTGAGTGTGAGGG-3′
*CCL2/MCP-1*	F: 5′-CTGTCTCAGCCAGATGCAGTTAA-3′
	R: 5′-AGCCGACTCATTGGGATCAT-3′
*IL-1β*	F: 5′-CTGGTACATCAGCACCTCTCAA-3′
	R: 5′-GAGACTGCCCATTCTCGACAA-3′
*IL-2*	F: 5′-ACAAGAATCTGAAACTCCCC-3′
	R: 5′-GAGATGATGCTTTGACAGATGG-3′
*IL-6*	F: 5′-GCCACTGCCTTCCCTACTTCA-3′
	R: 5′-GACAGTGCATCATCGCTGTTCA-3′
*IL-8*	F: 5′-CATTAATATTTAACGATGTGGATGCGTTTCA-3′
	R: 5′-GCCTACCATCTTTAAACTGCACAAT-3′
*PPAR-α*(h)	F: 5′-TCGACTCAAGCTGGTG-3′
	R: 5′-TTCCTGAGAGGATGACCC-3′
*CPT-1* (h)	F: 5′-GCCCATGTTGTACAGCTTCCA-3′
	R: 5′-AGTCTTCTTCCTTCATCAGTGGC-3′
*FXR* (h)	F: 5′- TGGACTCATACAGCAAACAGAGA -3′
	R: 5′- GTCTGAAACCCTGGAAGTCTTTT -3′
*LXR*	F: 5′-TCAGCATCTTCTCTGCAGACCGG-3′
	R: 5′- TCATTAGCATCCGTGGGAACA -3′
*TGR5*	F: 5′- CTGGCCCTGGCAAGCCTCAT -3′
	R: 5′- CTGCCATGTAGCGCTCCCCGT -3′
*β-actin* (h)	F: 5′-ACTATCGGCAATGAGCG-3′
	R: 5′-GAGCCAGGGCAGTAATCT-3′

(h) indicated that the primer is used for the detection of human gene.

### RNA Interference

RNA interference was performed as described previously [23]. Briefly, cultured cells were seeded into 6-well plates. After reaching 50% confluency, the cells were transfected with human PPAR-α or TGR5 siRNA (Santa Cruz Biotechnology) using Lipofectamine 2000 according to the manufacturer’s instructions. In the control group, cells were transfected with scrambled siRNA (Santa Cruz Biotechnology).

### Western Blotting

The detailed protocol of whole tissue or HL-7702 cells protein extracting and Western blot in our laboratory was described previously [24]. The nuclear protein from the fresh liver tissues or cultured cells was extracted using a Nuclear and Cytoplasmic Protein Extraction Kit (Beyotime Institute of Biotechnology). Antibodies used for protein detecting were bought from the indicated sources, *β*-actin, and carnitine palmitoyltransferase (CPT)-1 (Santa Cruz Biotechnology, CA), peroxisome proliferator-activated receptor (PPAR)-α, PPAR-γ and sterol regulatory element-binding protein (SREBP)-1c (Abcam, Cambridge, UK), fatty acid translocase (FAT/CD36) and acyl-CoA: diacylglycerol acyltransferase (DGAT)-1 (Life Span, Seattle), histone H3, AMP-activated protein kinase (AMPK) and phosphorylated-AMPK (Cell Sinaling, Boston). Experiments were replicated at least three times, and a representative blot is shown.

### Tissues Lipids Detection

Total lipids (TG, TC, and FFA) were extracted from tissues (liver and muscle) and measured using the method as described [25].

### Statistical Analysis

One-way analysis of variance (ANOVA), followed by the post-hoc test, was performed to analyze the data with SPSS software (version 16; Beijing Stats Data Mining, Beijing, China). Data were expressed as means ± S.E.M., and *P* values less than 0.05 were considered statistically significant.

## Results

### UA Supplementation Reversed HFD-induced Fatty Liver and Liver Injury

**Figure 1:**
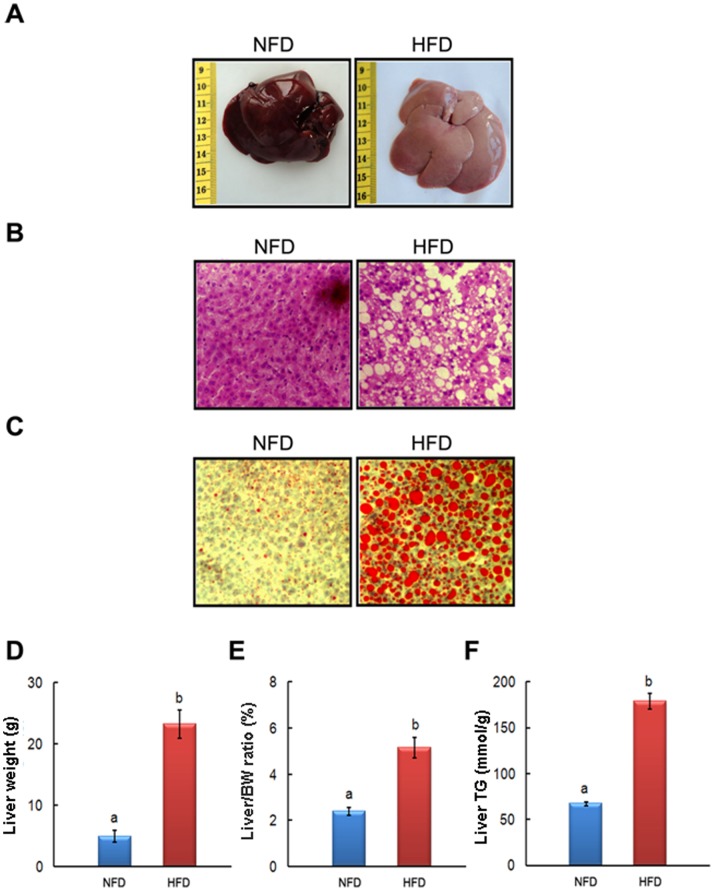
HFD-induced obese NAFLD rat model. The representative photographs and biochemical index were presented as follow: (A) liver morphological photographs, (B) H&E staining photomicrographs of the liver section (100×), (C) Oil Red O staining photomicrographs of the liver section (100×), (D) Liver weight, (E) relative weight of the liver, and (F) Liver triglyceride. Values are means ± SEM (NFD, n = 13; HFD, n = 70). The values with different superscripts are significantly different at *P*<0.05.

**Figure 2:**
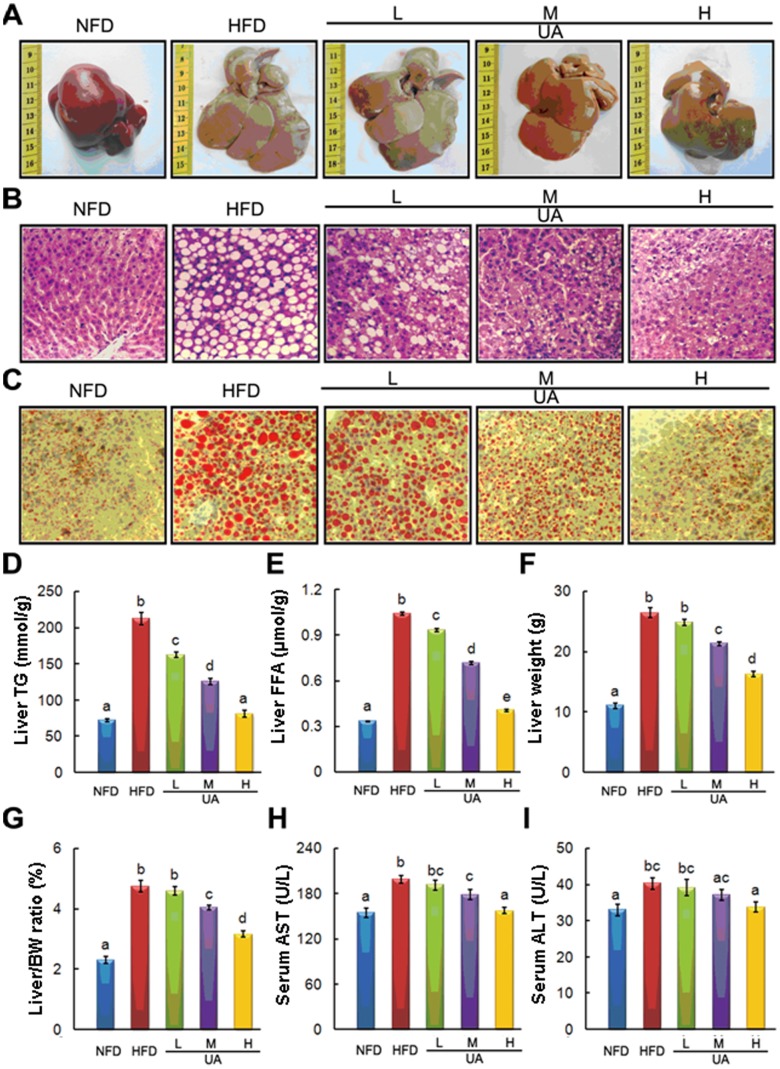
Effects of UA on the liver histology, hepatic lipids, and serum ALT and AST. The representative photographs and biochemical index were presented as follow: (A) Morphological photographs, (B) H&E staining photomicrographs of the liver section (100×), (C) Oil Red O staining photomicrographs of the liver section (100×), (D) Liver triglyceride, (E) Liver FFA, (F) Liver weight, (G) The ratio between liver and body weight, (H) Serum AST, and (I) Serum ALT. The values with different superscripts are significantly different at *P*<0.05. All the groups contain 10 animals (n = 10).

Obese NAFLD rat model was successfully established after 8 weeks HFD feeding. Body and liver weight along with serum and liver TG contents in HFD-fed rats were markedly increased compared to NFD group (Table 3 and Figure 1). After another 6 weeks UA treatment, the pathological alterations of livers from different groups were firstly evaluated by morphologic and histological (HE and Oil Red O staining) examination. Long-term HFD feeding significantly increased the size and lighted the color of liver (Figure 2A), and induced massive hepatic steatosis (Figure 2B, C). UA supplementation obviously reversed HFD-induced adverse changes mentioned above in a dose-dependent manner. The hepatic lipids contents test also confirmed that UA significantly reduced HFD-induced liver fat accumulation (Figure 2D, E). No difference of TC in liver was observed among those groups (data not shown). Beside, HFD-induced increase in liver weight and liver/body weight ratio were significantly alleviated by M- and H-UA supplemented to HFD (Figure 2F, G). Further, UA reversed HFD-induced liver injury indicated by the significant declining of circulating liver enzymes level, including AST and ALT (Figure 2H, I).

**Table 3:** Body weight and serum analysis in HFD-induced NAFLD rat model.

	NFD (n = 13)	HFD (n = 44)
Body weight (g)	409.70±4.9^a^	453.21±4.5^b^
GLU (mmol/l)	4.97±0.65^a^	4.99±0.61^a^
TG (mmol/l)	0.86±0.11^a^	1.18±0.27^b^
TC (mmol/l)	1.74±0.38^a^	1.72±0.30^a^
HDL-C (mmol/l)	1.13±0.09^a^	1.08±0.11^a^
LDL-C (mmol/l)	0.44±0.03^a^	0.49±0.06^b^
FFA (mmol/l)	1.20±1.19^a^	1.42±1.49^b^

### UA Reduced HFD-induced Body Weight and Fat/body Weight Ratio Increase

**Figure 3:**
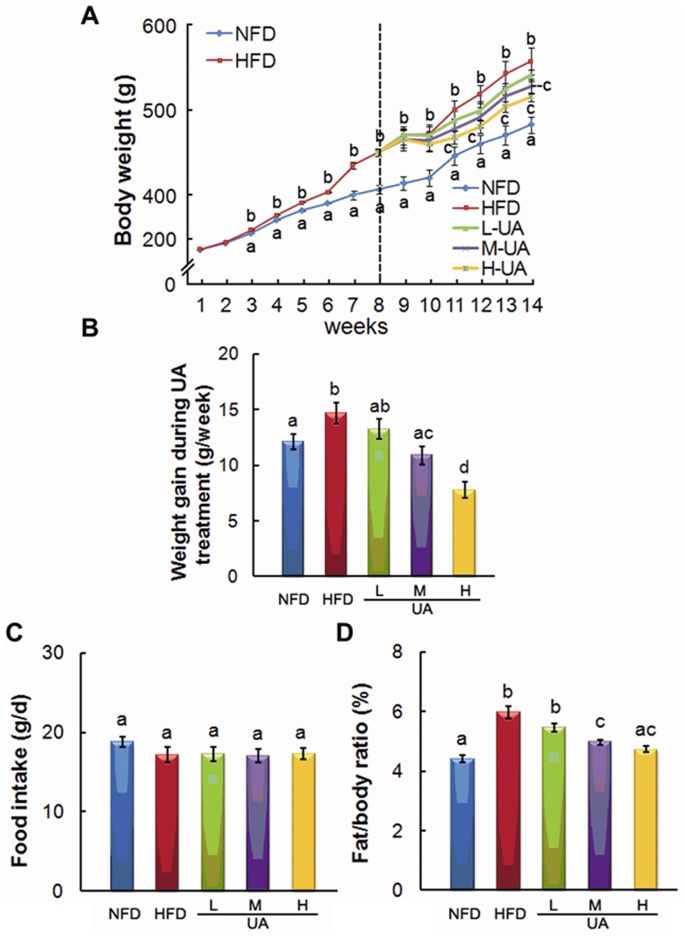
Effects of UA treatment on the characterization of the NAFLD rat. The effects of UA on the changes in body weight (A), body weight gain (B), food intake (C), and relative fat content (including perirenal and epididymal aidpose tissue, D) were detected. The values with different superscripts are significantly different at *P*<0.05. All the groups contain 10 animals (n = 10).

Dynamic change of body weight was measured during 14 weeks feeding period, and body weight gain was obtained by calculating the average change during UA treatment. M- and H-UA supplement significantly decreased HFD-induced increase of body weight at week 14 (Figure 3A), and HFD-induced weight gain was also alleviated by UA (Figure 3B). No difference of food intake was observed among experimental groups during the treatment (Figure 3C). Besides, fat/body weight ratio was markedly decreased by M- and H-UA compared with HFD group (Figure 3D).

### UA Alleviated HFD-induced Adverse Changes in Serum Biochemical Parameters

Serum TG, FFA and LDL-C, but not TC and HDL-C were significantly elevated in HFD rats compared to that in NFD rats. All the three supplemental dose of UA significantly decreased HFD-induced increase of serum TG, FFA and LDL-C in a dose-dependent manner (Table 4). Serum β-hydroxybutyrate in UA-treated groups was markedly elevated compared to that in HFD group (Table 4). Besides, UA treatment significantly alleviated HFD-induced insulin resistance by lowering insulin level and improving insulin resistance index (HOMA-IR), and decreasing leptin and elevating adiponectin levels in serum (Table 4).

**Table 4:** Serum analysis of ursolic acid treatment.

	NFD	HFD		L-UA	M-UA	H-UA
**Serum biochemical indicators (mmol/L)**						
TG	1.04±0.04 ^a^	1.21±0.03 ^b^		1.02±0.03 ^ac^	0.98±0.03 ^ac^	0.95±0.02 ^c^
TC	1.62±0.06^ a^	1.72±0.05 ^a^		1.62±0.04^ a^	1.62±0.05^ a^	1.61±0.06^ a^
HDL-C	1.13±0.04^ a^	1.03±0.04^ a^		1.01±0.03^ a^	1.04±0.04^ a^	1.10±0.04^ a^
LDL-C	0.43±0.02 ^a^	0.50±0.02 ^b^		0.48±0.02^ a^	0.42±0.02 ^a^	0.41±0.02 ^a^
FFA	1.19±0.02 ^a^	1.44±0.03 ^b^		1.23±0.02 ^a^	1.12±0.02 ^a^	1.00±0.02 ^c^
β-hydroxybutyrate (mmol/l)	0.92±0.03 ^a^	0.68±0.04 ^b^		0.91±0.04 ^a^	1.06±0.12^ a^	1.23±0.04 ^c^
**Insulin resistance relative markers**						
Insulin (ng/ml)	0.59±0.01 ^ad^	0.95±0.02 ^b^		0.80±0.02 ^c^	0.61±0.02 ^a^	0.54±0.02 ^d^
GLU (mmol/l)	5.00±0.27^ a^	5.07±0.16^ a^		5.03±0.25^ a^	4.94±0.24^ a^	4.92±0.23^ a^
HOMA-IR	8.38±0.30 ^a^	13.81±0.35 ^b^		11.50±0.23 ^c^	8.40±0.20 ^a^	7.74±0.27 ^a^
Leptin (ng/ml)	2.85±0.07 ^a^	7.78±0.06 ^b^		5.95±0.05 ^c^	3.70±0.08 ^d^	3.06±0.06 ^e^
Adiponectin (mg/l)	4.92±0.07 ^a^	2.27±0.08 ^b^		2.24±0.05 ^b^	3.23±0.06 ^c^	4.25±0.05 ^d^
**Inflammatory markers (pg/ml)**						
TNF-α	24.21±2.52 ^a^	64.70±3.37^b^		51.59±3.08 ^c^	37.01±1.91^d^	23.63±2.95 ^a^
CCL2/MCP-1	28.11±1.43 ^a^	48.62±1.04 ^b^		47.13±1.10 ^b^	42.84±1.17 ^c^	30.29±1.05 ^a^
IL-6	13.08±2.53 ^a^	42.45±3.51 ^b^		38.92±2.34 ^b^	31.15±1.71 ^c^	17.49±2.84 ^a^
**Oxidative stress markers**						
SOD (U/ml)	18.90±0.74 ^a^	12.80±0.80 ^b^		14.10±0.90 ^b^	15.90±0.64 ^b^	17.90±0.74 ^a^
MDA (nmol/ml)	8.73±0.94 ^a^	18.59±1.40 ^b^		14.04±1.00 ^c^	10.94±1.13 ^a^	9.91±1.08 ^a^
CAT (U/ml)	5.14±0.28 ^ad^	3.21±0.14 ^b^		3.87±0.25 ^c^	4.65±0.09 ^d^	5.50±0.10 ^a^
GSH-PX (U/L)	754.39±17.53 ^a^	300.45±21.07 ^b^		371.97±15.88 ^c^	556.26±25.97 ^d^	713.67±11.69 ^a^

The values with different superscripts are significantly different at P<0.05 (n = 10).

### UA Improved the Expression of TG and FFA Associated Genes

**Figure 4:**
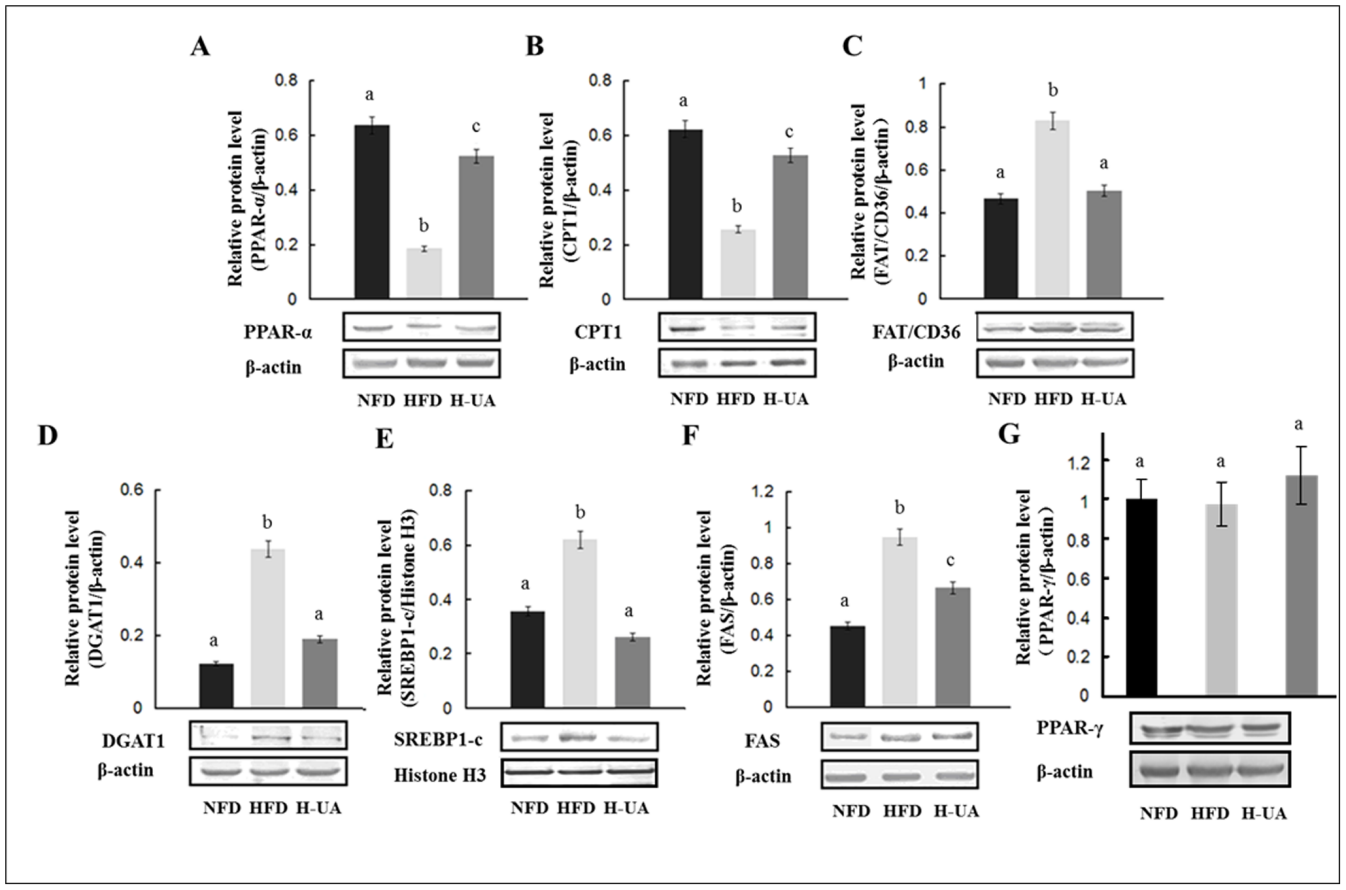
Effects of UA treatment on the liver relative protein content of the experimental rat. Liver relative protein content of (A) PPAR-α, (B) CPT1, (C) FAT/CD36, (D) DGAT1, (E) Mature form of SREBP-1c (∼68 KD) in the nucleus, (F) FAS and (G) PPAR-γ. The values with different superscripts are significantly different at *P*<0.05. All the groups contain 10 animals (n = 10), and a representative blot is shown.

**Figure 5:**
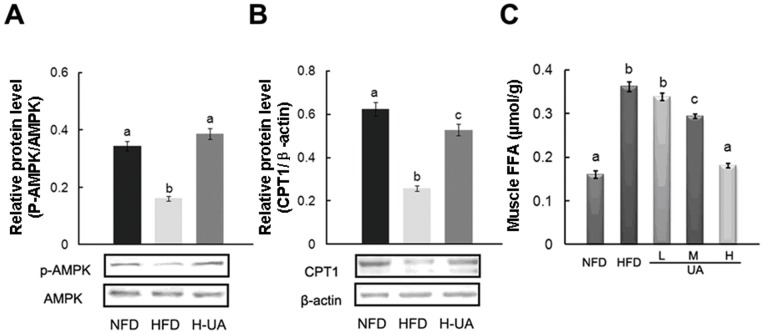
Effects of UA treatment on the muscle and the possible molecular mechanisms. Muscle relative protein content of (A) p-AMPK and (B) CPT-1. (C) Muscle FFA. The values with different superscripts are significantly different at *P*<0.05. All the groups contain 10 animals (n = 10), and a representative blot is shown.

NAFLD was characterized by the disorder of the expression of a series of lipid metabolic genes, including the increase of DGAT, FAT/CD36, SREBP-1c, ACC, FAS and the decrease of PPAR-α, CPT-1 [26], [27]. In our study (showed in Table 5 and Figure 4), UA treatment significantly reversed HFD-induced decrease in liver PPAR-α at both mRNA and protein levels. No significant change was observed on the protein expression of PPAR-γ in UA-treated group compared to that in HFD group. Further, DGAT, the primarily enzyme of TG synthesis, was significantly down-regulated in both mRNA and protein levels by UA. HFD-induced increase in FAT/CD36, SREBP-1c, ACC, and FAS in mRNA and/or protein levels were markedly reversed by UA treatment. The expression of CPT-1 was significantly up-regulated in both mRNA and protein levels by UA. Besides, no significant changes were observed on the mRNA expression of farnesoid X receptor (FXR) and liver X receptor (LXR) in rat liver in the UA-treated groups compared to HFD group (Table 5). In skeletal muscle, phospho-AMPK and CPT-1 protein expression were increased by UA (Figure 5A, B). FFA contents in skeletal muscle were decreased in UA supplemental groups compared with that in HFD group (Figure 5C).

**Table 5:** Effects of ursolic acid on hepatic genes expression.

	NF	HF	L-UA	M-UA	H-UA
**TG metabolism relative genes**					
*PPAR-α*	1 ^a^	0.61±0.03 ^b^	0.90±0.08 ^a^	0.81±0.08 ^ab^	1.35±0.02 ^c^
*FAT/CD36*	1 ^a^	2.66±0.17 ^b^	2.00±0.22 ^bc^	1.91±0.33 ^c^	0.67±0.21 ^a^
*DGAT1*	1 ^a^	1.83±0.14 ^b^	0.85±0.08 ^a^	0.54±0.04 ^c^	0.29±0.04 ^c^
*DGAT2*	1 ^a^	1.58±0.07 ^b^	0.94±0.34 ^a^	0.93±0.30 ^a^	0.71±0.07 ^c^
*ATGL*	1 ^a^	0.56±0.01 ^b^	0.47±0.02 ^b^	1.19±0.05 ^c^	2.25±0.05 ^d^
*HSL*	1 ^a^	0.60±0.04 ^b^	0.75±0.07 ^b^	1.54±0.06 ^c^	2.90±0.11 ^d^
*FXR*	1 ^a^	0.76±0.02 ^b^	0.78±0.05 ^b^	0.74±0.10 ^b^	0.89±0.21 ^ab^
*LXR*	1 ^a^	1.38±0.05 ^b^	1.24±0.14^ b^	1.20±0.11^ b^	1.01±0.27 ^ab^
**FFA metabolism relative genes**					
*CPT-1*	1 ^a^	0.45±0.03 ^b^	0.40±0.04 ^b^	0.92±0.01 ^a^	2.02±0.30 ^c^
*SREBP-1c*	1 ^a^	1.35±0.05 ^b^	0.95±0.01 ^a^	0.91±0.01 ^a^	0.61±0.07 ^c^
*FAS*	1 ^a^	1.45±0.07 ^b^	0.92±0.06 ^a^	0.56±0.06 ^c^	0.37±0.10 ^c^
*ACC*	1 ^a^	1.29±0.05 ^b^	0.84±0.03 ^a^	0.79±0.10 ^a^	0.49±0.12 ^c^
**Inflammatory factors**					
*TNF-α*	1 ^a^	2.08±0.47 ^b^	1.01±0.13 ^a^	0.65±0.13 ^c^	0.55±0.17 ^c^
*CCL2/MCP-1*	1 ^a^	6.89±0.12 ^b^	0.93±0.11 ^a^	1.20±0.21 ^a^	0.43±0.11 ^c^
*IL-1β*	1 ^a^	1.87±0.11 ^b^	0.95±0.01 ^ac^	0.74±0.12 ^c^	0.76±0.01 ^ac^
*IL-2*	1 ^a^	1.40±0.11 ^b^	0.28±0.14 ^c^	0.16±0.03 ^c^	0.12±0.01 ^c^
*IL-6*	1 ^a^	1.21±0.17 ^ab^	1.02±0.16 ^a^	0.89±0.12 ^a^	0.86±0.15 ^a^
*IL-8*	1 ^a^	3.95±0.52 ^b^	3.39±0.42 ^bc^	1.70±0.55 ^a^	1.40±0.33 ^a^

The values with different superscripts are significantly different at *P*<0.05 (n = 10).

### Knockdown PPAR-α Inhibited the Anti-steatosis Role of UA *in vitro*

**Figure 6:**
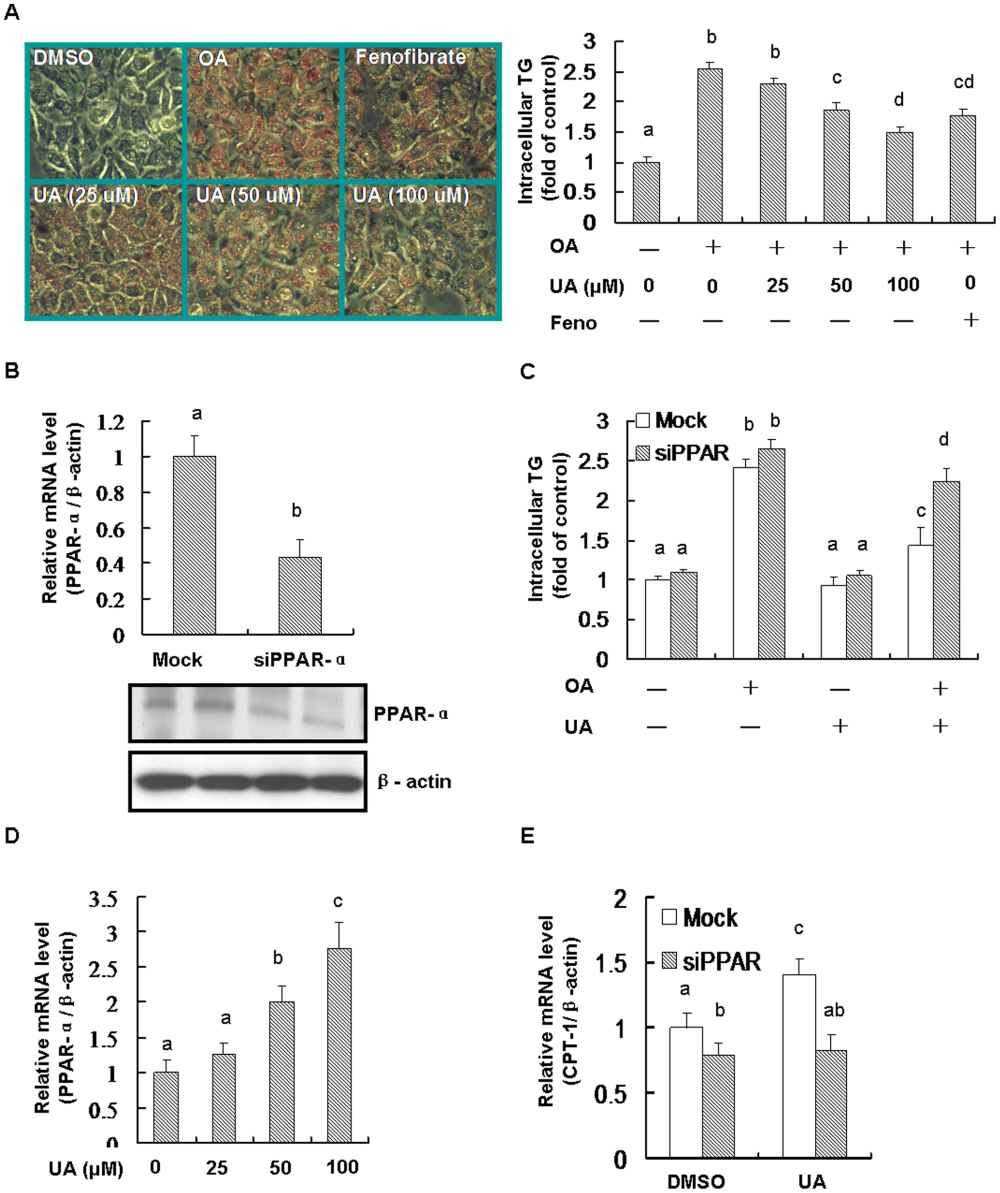
PPAR-α contributed to the anti-steatosis role of UA in HL-7702 cells. Equal (DMSO) was added into the medium for each group. Cells were seeded into 6-well plate and incubated with oleic acid (OA, 0.5 mM) for 24 h, and then OA was removed and the cells were treated with UA (100 µM, or as indicated) or fenofibrate (Feno, 100 µM) for another 24 h. For PPAR-α knockdown, siRNA was used according to the instructions as described in the Methods. (A) Cell were stained with oil red O and imaged by microscope. The intracellular TG was detected by triglyceride assay kit (Applygen Technologies Inc, Beijing, China) according to the manufacturer’s instructions. (B) The levels of mRNA and protein of PPAR-α were detected for testing the siRNA efficiency. (C) Knockdown PPAR-α inhibited the anti-steatosis role of UA and. (D) UA activated PPAR-α mRNA expression. (E) Knockdown PPAR-α blocked UA stimulated increase of CPT-1. Different alphabet indicates statistically significant differences (*P*<0.05). Data were expressed as means ± SD. All the experiments were repeated at least 3 times.

OA was widely used to build hepatic steatosis model *in vitro*. In this study, OA significantly elevated intracellular TG contents in human normally hepatic cells. OA-induced TG deposition was markedly reversed by UA in a dose-dependent manner. Fenofibrate, which was used as a PPAR-α agonist, was shown to reduce OA-induced steatosis (Figure 6A). The anti-steatosis role of UA was significantly inhibited after knocking down PPAR-α, indicating that PPAR-α pathway was involved in the protective function of UA (Figure 6B, C). UA was shown to strongly increase mRNA expression of PPAR-α in a dose-dependent manner (Figure 6D). Knockdown PPAR-α significantly blocked the elevated role of UA on mRNA expression of PPAR-α target gene CPT-1 (Figure 6E).

### Knockdown TGR5 did not Inhibited the Anti-steatosis Role of UA

**Figure 7:**
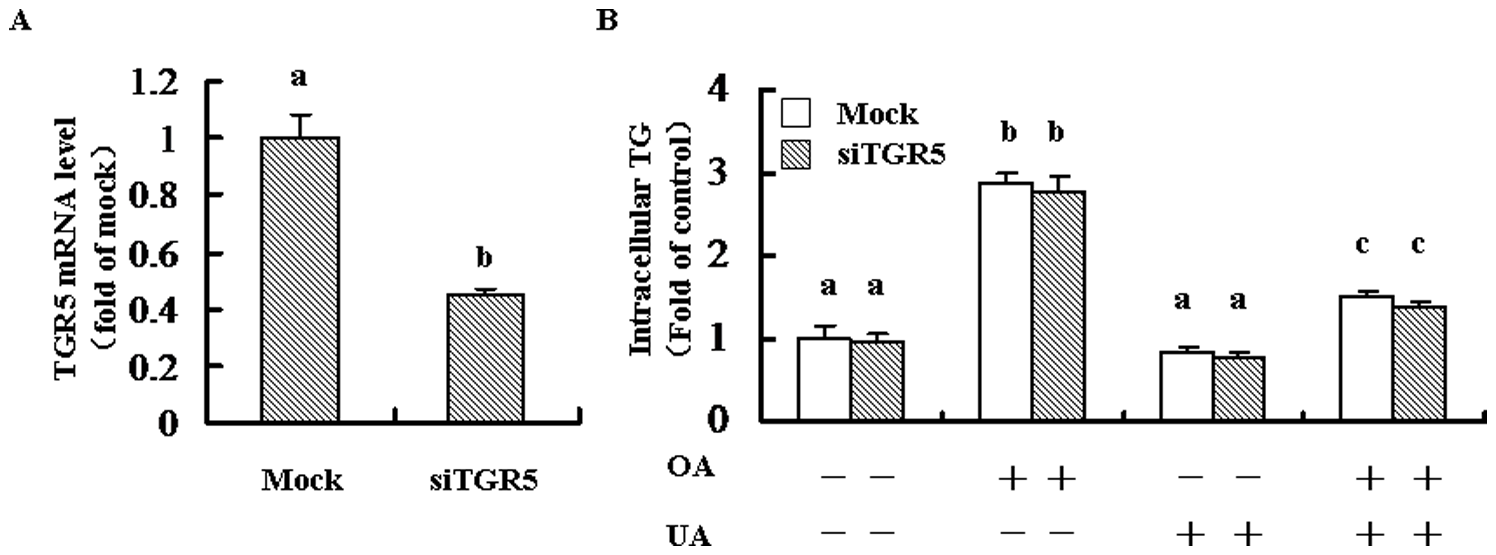
TGR5 did not contributed to the anti-steatosis role of UA in HL-7702 cells. Cells were seeded into 6-well plate and transfected with siRNA for TGR5 according to the instructions as described in the Methods. Cells were incubated with oleic acid (OA, 0.5 mM) for 24 h, and then OA was removed and the cells were treated with UA (100 µM) for another 24 h. Equal amount of DMSO was added was added into the medium for each group. (A) mRNA level of TGR5 was detected for the analysis of transfection efficiency. (B) The intracellular TG was detected by triglyceride assay kit (Applygen Technologies Inc, Beijing, China) according to the manufacturer’s instructions. Different alphabet indicates statistically significant differences (*P*<0.05). Data were expressed as means ± SD. All the experiments were repeated at least 3 times.

As shown in Figure 7A, using siRNA for TGR5 significantly knocked-down the expression of TGR5. As we expected, UA markedly reduced OA-induced accumulation of intracellular TG. However, knocking-down TGR5 did not alter the anti-steatosis role of UA (Figure 7B).

### UA Ameliorated HFD-induced Hepatic and Systemic Inflammation

UA supplement significantly decreased hepatic inflammatory factors mRNA expression, including *TNF-α*, *CCL2/MCP-1*, *IL-1β*, *IL-2*, *IL-6* and *IL-8* (Table 5). Serum levels of TNF-α, CCL2/MCP-1, IL-6 were also lowered by UA compared with that in HFD rats (Table 4).

### UA Decreased HFD-induced Oxidative Stress

Anti-oxidative ability of UA was also detected by analyzing serum SOD, MDA, CAT, and GSH-PX levels. The results showed that HFD-induced adverse variations in these markers were significantly reversed by UA treatment (Table 4).

## Discussion

Using a well-accepted HFD-induced NAFLD rat model, we reported the therapeutic role of UA for the first time on alleviating hepatic steatosis and liver injury, and further improving metabolic disorders, including serum lipid disorder, insulin resistance, inflammation and oxidative stress.

HFD-induced hepatic steatosis rat model is comprehensively used in the preventing and curing of NAFLD, since the great association between NAFLD and obesity. The model can be established as early as 4 weeks, characterized by significant increase of lipid accumulation in the liver and weight gain [28]. Both macroscopic and microscopic results from our study showed severe hepatic steatosis and obesity in HFD fed rats after 8 weeks, demonstrating the successful establishment of obese NAFLD rat model. UA is generally considered to be safe and have minimal adverse effect. Neither mortality nor any signs of toxicity with orally administrating a single dose as high as 2,000 mg/Kg was observed in acute toxicity study in mice [29]. In our study, no toxic effect on experimental animals was observed in our administrating dose of UA (about 200 mg/Kg body weight day in H-UA group).

PPAR-α is a well accepted potential therapeutic target for its pivotal role in the regulation hepatic lipid metabolism by stimulating the transcription of PPAR-α regulated genes [6], [30], such as CPT-1, the rate-limiting enzyme for the transport of long-chain fatty acids across the membrane of mitochondria [7]. PPAR-α defective mice failed to induce fatty acid oxidation in liver and developed severe steatohepatitis immediately after birth [5]. In the state of NAFLD, hepatic PPAR-α was significantly decreased [30]. Activating PPAR-α was shown to prevent the development of steatosis [6], [30]. In our study, UA obviously reversed HFD-induced down-regulation of PPAR-α in both mRNA and protein levels, which was accordant with the *in vitro* study, in which UA was regarded as a potential PPAR-α agonist [31]. To further test the beneficial role of PPAR-α on anti-steatosis, human normally hepatic cell line HL-7702 was employed. Our results indicated that UA significantly stimulated mRNA expression of PPAR-α, and decreased OA-induced intracellular TG deposition. Moreover, the anti-steatosis role of UA was lost after knocking down PPAR-α by siRNA. The UA-stimulated increase in mRNA expression of CPT-1, the target gene of PPAR-α, was also inhibited by silencing PPAR-α. These results indicated that PPAR-α pathway contributed to the therapeutic role of UA on hepatic steatosis.

Besides, both PPAR-α and PPAR-γ could be induced by the activation of nuclear receptor FXR in liver cells [32], [33]. We therefore, proposed that whether UA-induced activation of PPAR-α in a FXR involved pathway. However, UA supplementation did not increase the expression of FXR at mRNA level compared to that in HFD group. Meanwhile, the protein expression of PPAR-γ in rat liver was also not affected by UA treatment. Taken together with a recent study, which also reported that UA was not a FXR inducer [34], we proposed that FXR was not participating in the UA-induced PPAR-α. We also tested the potential role of nuclear transcription factor LXR on the anti-steatosis effect of UA. However, UA supplementation did not alter HFD-induced increase of LXR at mRNA level in experimental animal liver. In vitro, UA treatment also did not affect nuclear LXR protein expression with or without LXR agonist T0901317 pre-treatment (data not shown). These results implied that LXR was independent from the anti-steatosis role of UA. TGR5 is a recently identified plasma membrane-bound, G protein-coupled receptor for bile acids, which is ubiquitously expressed, with high expression in liver, intestine, brown adipose tissue, and spleen [35]. Recently, UA was regarded as a TGR5 activator and activating hepatic TGR5 was shown to improve NAFLD in obese db/db mice [34], [36]. We, therefore, proposed that whether the anti-steatosis role of UA was exerted via a TGR5 involved pathway. However, our data demonstrated that knocking-down TGR5 did not affect the protective role of UA, which excluded the involvement of TGR5.

Based on the two-hit hypothesis [4], reducing liver TG accumulation is a conceivable strategy to treat NAFLD in the early stage through stimulating TG hydrolysis or decreasing TG synthesis. Inhibition of DGAT showed a marked reduction in hepatic TG in HFD-induced obese mice [37]. In this study, UA significantly reduced expressions of DGAT in both mRNA and protein levels, which might also be involved in the hepatic TG deposition lowering role of UA. Although DGAT was regarded as a target gene of PPAR-alpha in some reports, in our study, HFD-induced the increase of DGAT was obviously reduced by UA supplementation, which suggested that UA-regulated DGAT was in a PPAR-alpha independent pathway. The similar situation has also been found when using PPAR-alpha agonist fenofibrate [38] or some compounds, such as osthol [39] and mangiferin [25]. It is interesting to address the potential mechanisms by which UA down-regulated DGAT in the future study. FFA, the primary product of TG enzymolysis, is an acknowledged risk factor for NAFLD, insulin resistance and inflammation. In our study, UA reversed HFD-induced serum FFA increase. We firstly excluded FFA restored into adipose tissue as TG, because the body fat content in UA-treatment group was reduced compared to that in HFD group. This also could be explained by our previous study that UA enhanced adipocytes lipolysis via stimulating ATGL and HSL [14]. In addition, ACC and FAS, the rate-limiting enzyme for FFA synthesis, along with their transcriptional factor SREBP-1c, are increased in the processing of NAFLD [40]. Activation of PPAR-α reduced the expression SREBP-1c [38], and thus blocked endogenous FFA synthesis through inhibiting ACC and FAS [41]. Our results showed that the infaust change of SREBP-1c, ACC and FAS were markedly ameliorated by UA treatment, indicating that the inhibition of FFA synthesis was also involved in the anti-steatosis role of UA. Further, CPT-1, which was positive regulated by PPAR-α, was declined in NAFLD state [7], [30], [42]. Activating PPAR-α increased the expression of CPT-1 and thus facilitated lipid oxidation and decreased lipid deposition [7], [30]. In this study, CPT-1 was significantly up-regulated in both liver and skeletal muscle by UA, which was benefit for FFA transport into mitochondrial for further oxidation. Although UA-induced beta-oxidation was not directly detected in our study, we proposed that FFA catabolism was induced by UA, because FFA levels in both hepatic and skeletal muscular, along with that in serum, were reduced dramatically in UA-treated group. Besides, β-hydroxybutyrate (ketone body), the main alternative energy substrate to glucose, is generated by hepatic fatty acid oxidation in liver when plasma glucose and insulin are low [43]. UA supplementation increased the levels of β-hydroxybutyrate in blood in our study, indicating that UA could increase FFA oxidation. In mice, chronic treatment with UA significantly increased energy expenditure during both dark and light cycles without significantly altering spontaneous activity [16]. Further, hepatic FAT/CD36 was accordingly down-regulated by UA, which was probably due to the reduction of systemic FFA.

Inflammation and insulin resistance, which are closely accompanied with lipid metabolism, are implicated in the pathogenesis of NAFLD and the progression of non-alcoholic steatohepatitis (NASH), and further fibrosis and cirrhosis [4], [44]. Several cytokines, including TNF-α, IL-6, and CCL2/MCP-1, have been incriminated to play an essential pathogenic role in NASH [4], [45], [46]. In the present study, UA significantly alleviated systemic and hepatic inflammatory evidenced by the reduction of HFD-induced increase of TNF-α, IL-6, and CCL2/MCP-1 in serum and hepatic TNF-α, IL-1, IL-2,IL-6, IL-8,and CCL2/MCP-1 mRNA expressions. Moreover, accumulating evidence from both *in vitro* and animal studies have attributed anti-inflammatory activity to PPAR-α up-regulation [7]. Administrating NASH mice with PPAR-α agonist apparently reversed steatohepatitis [47], demonstrating that the anti-inflammatory effect of UA in our study was mediated, at least partially, by PPAR-α involved pathway. Inflammatory cytokines, such as TNF-α, IL-6 and others, have been shown to promote insulin resistance and liver inflammation [48], [49]. In addition, leptin and adiponectin, with received abilities of both insulin sensitization and anti-inflammation, have been found to affect liver fibrogenesis [48], [50]. Although the exact anti-pathogenic role of leptin and adiponectin has not been elucidated, disadvantage alteration of their levels has been observed in patients with NASH [48], [51]. Results in this study indicated that HFD-induced the increase of leptin and the decrease of adiponectin were recovered by UA supplementation. Along with the improving of HOMA-IR, these results strongly suggested the insulin sensitization of UA in the processing of NAFLD.

Hepatic lipid accumulation and/or it induced inflammation and insulin resistance, act as a powerful oxidative stressors, causing overproduction of reactive oxygen species (ROS) and depleting the concentration of natural cellular antioxidants [40]. Increased production of ROS has been seen in animal models of NASH [52]. Besides, hepatic antioxidant capacity was reduced in patients with NAFLD [53]. In this study, UA exhibited obviously anti-oxidative ability through improving hepatic lipid accumulation. HFD-induced harmful changes in detected antioxidant enzymes were markedly relieved by UA.

In summary, we penetratingly reported the curative role of UA on NAFLD with obesity for the first time. Moreover, our results indicated that UA reversed HFD-induced hepatic steatosis. Further, this beneficial effect of UA was associated with a PPAR-α participated pathway. Inflammation, insulin resistance and oxidation stress conditions were accordingly ameliorated with the improvement of lipid metabolism. This is the first study that recommends UA, a safe natural phytochemicals, for the treatment of NAFLD.
