# Correction: Therapeutic Role of Ursolic Acid on Ameliorating Hepatic Steatosis and Improving Metabolic Disorders in High-Fat Diet-Induced Non-Alcoholic Fatty Liver Disease Rats

**DOI:** 10.1371/journal.pone.0092364

**Published:** 2014-03-07

**Authors:** The PLOS ONE Staff

The second and third authors are listed out of order. The correct author order is: Songtao Li, Xilu Liao, Fanyu Meng, Yemei Wang, Zonxiang Sun, Fuchuan Guo, Xiaoxia Li, Man Meng, Ying Li, Changhao Sun.

## References

[1] Li S, Meng F, Liao X, Wang Y, Sun Z, et al (2014) Therapeutic Role of Ursolic Acid on Ameliorating Hepatic Steatosis and Improving Metabolic Disorders in High-Fat Diet-Induced Non-Alcoholic Fatty Liver Disease Rats. PLoS ONE 9(1): e86724 doi:10.1371/journal.pone.0086724 24489777 10.1371/journal.pone.0086724 PMC3906058

